# DPP-4 inhibition enhanced renal tubular and myocardial GLP-1 receptor expression decreased in CKD with myocardial infarction

**DOI:** 10.1186/s12882-019-1243-z

**Published:** 2019-03-01

**Authors:** Seung Jung Kim, Soon Kil Kwon, Hye-Young Kim, Sun Moon Kim, Jang-Whan Bae, Joong-Kook Choi

**Affiliations:** 1 Department of Internal Medicine, College of Medicine, Chungbuk National University, 1 Chungdaero, Seowongu, Cheongju, Chungbuk 28644 Republic of Korea; 2 Department of Biochemistry, College of Medicine, Chungbuk National University, Cheongju, Republic of Korea

**Keywords:** Chronic kidney disease, Myocardial infarction, GLP-1 receptor, DPP-4 inhibitor

## Abstract

**Background:**

Chronic kidney disease (CKD) is strongly associated with cardiovascular disease and is a significant risk factor for increased morbidity and mortality. In contrast, GLP-1 receptor (GLP-1R) activation has been shown to confer both renal and cardiovascular protection, though its relationship with CKD and CKD with myocardial ischemia/reperfusion (MI/R) remains poorly understood. Here, we investigated changes in renal and myocardial GLP-1R expression in the CKD rat model with MI/R.

**Methods:**

Male Sprague Dawley rats with 5/6 nephrectomy were used as a rat model of CKD and CKD with MI/R. For myocardial ischemia, the left coronary artery was ligated and released for 30 min 1 week after 5/6 nephrectomy. Dipeptidyl-peptidase 4 (DPP-4) inhibitors were administered orally with linagliptin once daily for 8 weeks. Renal cortical and myocardial GLP-1R expression were measured via immunohistochemistry and western blot analysis.

**Results:**

DPP-4 activity was increased in CKD. Western blot density of GLP-1R in renal cortex extracts revealed increased abundance 2 weeks after 5/6 nephrectomy, followed by a decrease at 8 weeks. In contrast, CKD and CKD with MI/R rats showed decreases in renal and cardiac expression of GLP-1R; these effects were attenuated in rats treated with linagliptin.

**Conclusions:**

In CKD with MI/R, linagliptin attenuated renal injury and increased renal and myocardial GLP-1R expression. These data suggest that activation of renal and myocardial GLP-1R expression may provide both cardio- and renoprotective effects.

**Electronic supplementary material:**

The online version of this article (10.1186/s12882-019-1243-z) contains supplementary material, which is available to authorized users.

## Background

Chronic kidney disease (CKD) is common problem in the elderly with ~ 13% of the population affected by some form of CKD [1]. The presence of CKD, whether manifested as albuminuria or reduced glomerular filtration rate, has been identified as an independent cardiovascular risk factor and is associated with higher mortality [2]. Diabetes is the most common cause of CKD, and control of diabetes and its associated vascular complications can ameliorate the progression of CKD [3].

Cardiovascular disease is the most common cause of death in CKD [4], with a prevalence 10 times higher than that of the general population [5]. Numerous interventions have been developed that target known cardiovascular risk factors, with a goal of reducing mortality in CKD patients. Targeting of left ventricular problems associated with coronary artery disease [6], as well as prevention of vascular calcification and coronary atherosclerosis, have proven effective for reducing the risk of cardiovascular events in CKD patients [7, 8].

Glucagon-like peptide-1 (GLP-1) is an incretin hormone that enhances insulin secretion, and GLP-1 agonists are used for glycemic control of type 2 diabetes [9]. GLP-1 plays a role in cardiovascular protection by increasing myocardial insulin sensitivity and vascular endothelial cell protection [10]. GLP-1 receptors (GLP-1R) are expressed in the pancreas, heart, brain, lungs, renal proximal tubules, and glomeruli [11, 12]. GLP-1 and dipeptidyl-peptidase 4 (DPP-4) inhibitors can protect against proteinuria and renal disease progression. However, the precise role of renal GLP-1R in kidney protection remains to be unveiled, and the changes in GLP-1R that occur during renal impairment are unknown.

We aimed to determine the association between changes in renal tubular GLP-1R expression and CKD progression, as well as to investigate any changes in GLP-1R after DPP-4 inhibition. Analyses were performed in vitro using cultured renal tubular and myocardial cells isolated from CKD rats with myocardial ischemia/reperfusion (MI/R). We also investigated myocardial changes in GLP-1R expression, as well as extracellular signal-regulated kinase (ERK1/2) and B cell lymphoma-2 (Bcl-2) protein levels in ischemic injury before and after DPP-4 inhibition.

## Methods

### Animal groups, clinical parameters, urine, and blood test

**Fig. 1:**
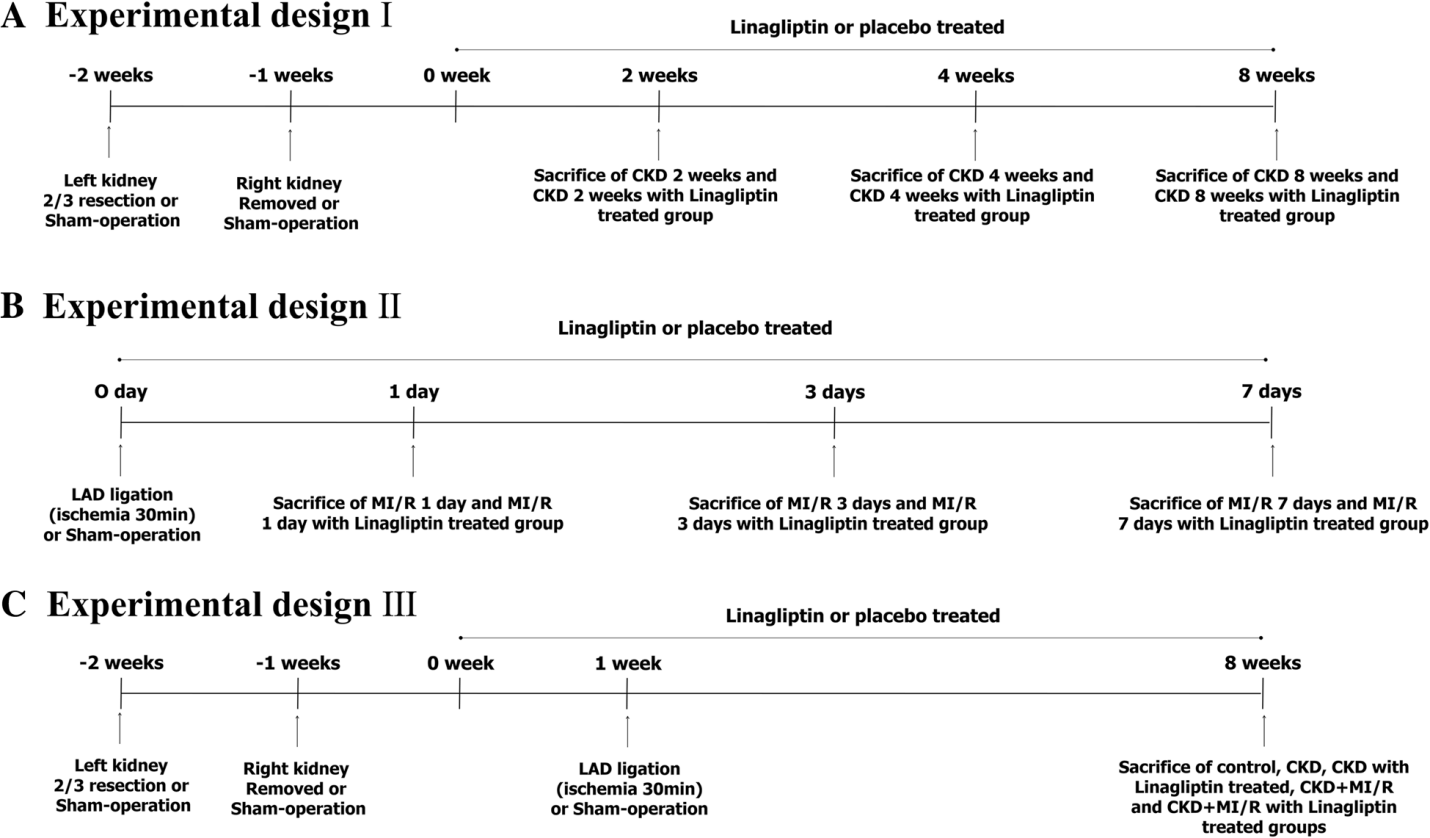
Experimental design. CKD, 5/6 nephrectomy; MI/R, myocardial ischemia reperfusion; CKD + MI/R, 5/6 nephrectomy with myocardial ischemia reperfusion. (*n* = 6 each). **a** Experimental design I **b** Experimental design II **c** Experimental design III

All animal experiments were approved by the Institutional Animal Research Board of Chungbuk National University (CBNUA-1051-17-02). Male Sprague Dawley rats (*n* = 90) approximately 200 to 250 g weight (Daehan Biolink, Chungbuk, Korea) were randomized into 15 groups with 6 animals in each group: control, CKD 2, 4, and 8 weeks with/without linagliptin, MI-R 1, 3, and 7 days with/without linagliptin, CKD 8 weeks + MI-R with/without linagliptin. We established three experimental designs for CKD, acute MI-R, and CKD with MI-R (Fig. 1). Sample size was calculated by Mead’s resource equation, and we added animals against incidental peri-operative animal death. Blood pressure was measured via a femoral arterial pressure monitoring sensor (MLT844, Memscap AS, Skoppum, Norway), which was supported by a bridge amp (ML112, AD Instruments, Sydney, Australia) and Powerlab/4sp (ML750, AD Instruments). Urine was collected, with volume measured for a 24 h period. Creatinine clearance was assessed via a metabolic cage with mineral oil treatment on the day of organ harvesting (the last day). Blood samples were obtained via femoral venous sampling, centrifuged, and stored at − 80 °C. Body weight, serum glucose, creatinine, and albumin levels were measured using a Nova Stat Profile M critical care analyzer (Nova Biomedical Co., Waltham, MA, USA) and compared between groups. The pH of fresh urine was measured with a pH meter (Orion 3 star plus, Thermo, Beverly, MA, USA).

### CKD rat model and DPP-4 inhibitor treatment

In all CKD groups, the lower and upper thirds of the left kidneys were resected, and after 1 week, the right kidney was removed (5/6 nephrectomy); a sham operation was performed in the control group. For general anesthesia, 50 mg/kg of tiletamine plus zolazepam (Zoletil) and 10 mg/kg of xylazine (Rompun) were mixed and injected into the thigh muscle. Rats were divided into seven groups: control, CKD 2, 4, 8 weeks with or without DPP-4 inhibitor-treated. All DPP-4 inhibitor-treated groups were orally administered 5 mg/kg of linagliptin (Trajenta, Boehringer-Ingelheim, Columbus, OH, USA) mixed in water, once daily for 8 weeks beginning the day after 5/6 nephrectomy.

### Myocardial infarction model in the CKD rat

Using the same anesthesia method described above, the left fourth intercostal space was dissected to expose the heart and an endotracheal catheter with rodent ventilator (model 683, Harvard Apparatus, South Natick, MA, USA) application was inserted. The left anterior descending branch of coronary artery (LAD) was then ligated with 6–0 silk for 30 min and released after ischemia (ischemia-reperfusion) via same method published by our college laboratory [13]. Acute GLP-1R changes among the groups were assessed 1, 3, and 7 days with or without DPP-4 inhibitor-treated after myocardial infarction. CKD-MI model were divided into five groups (*n* = 6 each): control, CKD 8 weeks, CKD 8 weeks with DPP-4 inhibitor (CKD-Lina), CKD 8 weeks with myocardial ischemia/reperfusion (CKD-MI), and CKD-MI with DPP-4 inhibitor (CKD-MI-Lina). For the myocardial infarction model, animals were treated with linagliptin for 8 weeks, after which the heart was harvested for immunoblotting and histochemical staining. The survival rate was analyzed by SPSS (SPSS 24.0; SPSS Inc., Chicago, IL, USA) and displayed by Kaplan-Meier survival curves. After the end of all experiments, animals were euthanized by ether inhalation without pain or stress which was based on the Institutional animal care and use committee (IACUC) standard operating guideline and approval (CBNUA-1051-17-02).

### Cell culture and hypoxic injury

Rat kidney proximal tubule (NRK-52E) cells (American Type Culture Collection, Manassas, VA, USA) were cultured in Dulbecco’s Modified Eagle Medium (DMEM; Welgene, Kyeongbuk, Korea) supplemented with 5% fetal bovine serum (FBS; Gibco, Grand Island, NY, USA), 100 U/mL of penicillin, and 100 mg/mL of streptomycin (Gibco). Primary cultures of cardiomyocytes (CMCs) were isolated by modification of reported protocols [14–16]. Obtained ventricles were washed three times with cold ADS buffer, chopped, and digested three times for 20 min with collagenase (0.5 mg/mL) and pancreatin (0.3 mg/mL). The resulting cells were collected and enriched by differential centrifugation through a discontinuous Percoll (Amersham Pharmacia Biotech, Piscataway, NJ, USA) gradient of densities 1.050, 1.062, and 1.082 g/mL [17]. The band which collected at the 1.062/1.082 density interface was collected and used as the source of CMCs. The CMCs were washed and suspended in DMEM supplemented with medium 199 (Welgene), 10% horse serum (Gibco), 5% FBS, 5000 U/L streptomycin, and 5000 U/L penicillin plus 10% (*v*/v), and seeded at a density of 5 × 10^5^ mononuclear cells/well on 2% gelatin (Sigma-Aldrich, St. Louis, MO, USA) coated on 60 mm Primarian 6-well plastic culture plates (Becton Dikenson and company, Hunt Valley, MD, USA). Media changes were initiated on day 1 via addition of serum free DMEM/M199 medium (4:1). NRK-52E cells and CMCs were incubated in a humidified atmosphere with 37 °C and 5% CO_2_. For hypoxic conditions, NRK-52E cells cultured 2, 6, 12, 24, and 48 h and CMCs cultured for 1, 3 and 5 days were placed in a GasPakTM pouch system (Becton Dickinson, Franklin Lakes, NJ, USA) in conditions of 1% O_2_ and 5% CO_2_.

### Serum DPP-4 enzyme activity

Serum DPP-4 enzyme activity was measured using the DPP-4 Activity Assay Kit (Biovision, Milpitas, CA, USA). Briefly, 50 μL of serum was diluted with 48 μL of DPP IV assay buffer and 2 μL substrate Gly-Pro-7-Amino-4-Methylcoumarin (AMC), and incubated at 37 °C for 30 min. The AMC density of the substrate was then measured by spectrophotometer at excitation and emission wavelengths of 360 and 460 nm, respectively.

### Western blot (WB) analysis

Renal and myocardial GLP-1R expression was measured in renal cortex and heart tissue by WB using specific antibodies (Bioss, Boston, MA, USA) with β-actin (Sigma-Aldrich) and glyceraldehyde 3-phosphate dehydrogenase (GAPDH, Young-In Frontier, Seoul, Korea) as a loading control. Proteins were extracted with protein extraction solution (PRO-PREP, Intron, Korea) and measured by spectrophotometer. Samples were loaded in 10% polyacrylamide-sodium dodecyl sulfate mini gels and transferred to polyvinylidene fluoride (PVDF) membranes. Membranes were blocked for 2 h in tris-buffered saline-0.1% plus Tween20 (TBS-T) containing 5% non-fat dry milk, treated with primary antibodies against GLP-1R, phospho ERK1/2, total ERK1/2, Bcl-2 (Santa Cruz Biotechnology, Santa Cruz, CA, USA), and β-actin for 2 h in TBS-T, followed by the secondary antibody (goat anti-rabbit IgG-horseradish peroxidase; Santa Cruz Biotechnology, Santa Cruz, CA, USA). Immunohistochemical staining was used to assess changes in renal tubular expression between groups, and to assess the effects of renal impairment and DPP-4 inhibition on protein expression. WB band densities were quantified using the Multi Gauge 3.1 program and expressed as a percentage relative to the control group.

### Kidney and heart tissue preparation and immunohistochemistry (IHC) and Masson’s trichrome stain

The kidneys and heart were harvested from all experimental and control groups. The renal cortex, outer medulla, and inner medulla were separated and fixed in 8% periodate-lysine-paraformaldehyde (PLP) solution for 8 h at room temperature, stored at 4 °C overnight, and embedded in paraffin. For IHC processing, tissues sections were rinsed in xylene to remove paraffin, then rehydrated using a gradient of 100 to 70% ethanol. Endogenous peroxidase activity was inhibited by treatment with 3% H_2_O_2_ at 4 °C for 45 min. Normal goat serum (Vector Laboratories, Inc., Burlingame, CA, USA) treated slides were exposed to primary antibodies at 4 °C overnight, then exposed to biotinylated goat anti-rabbit IgG (MACH2 Rabbit HRP Polymer; Concord, Biocare Medical, Burlingame, CA, USA) at room temperature for 30 min. After antibody exposure, heart tissue sections were treated with peroxidase 3, 3′-diaminobenzidine (DAB) substrate, and mounted after rinsing with xylene. These paraffin blocks were sectioned with 4 μm thickness and stained with Masson’s-Trichrome for the evaluation of extent of infarcted area.

### Statistical analyses

As each study group contained 6 animals, all data are presented as mean ± standard deviation. An independent sample *t*-test was used for comparing experimental and control groups (SPSS).

## Results

### Changes in clinical parameters in CKD

**Fig. 2:**
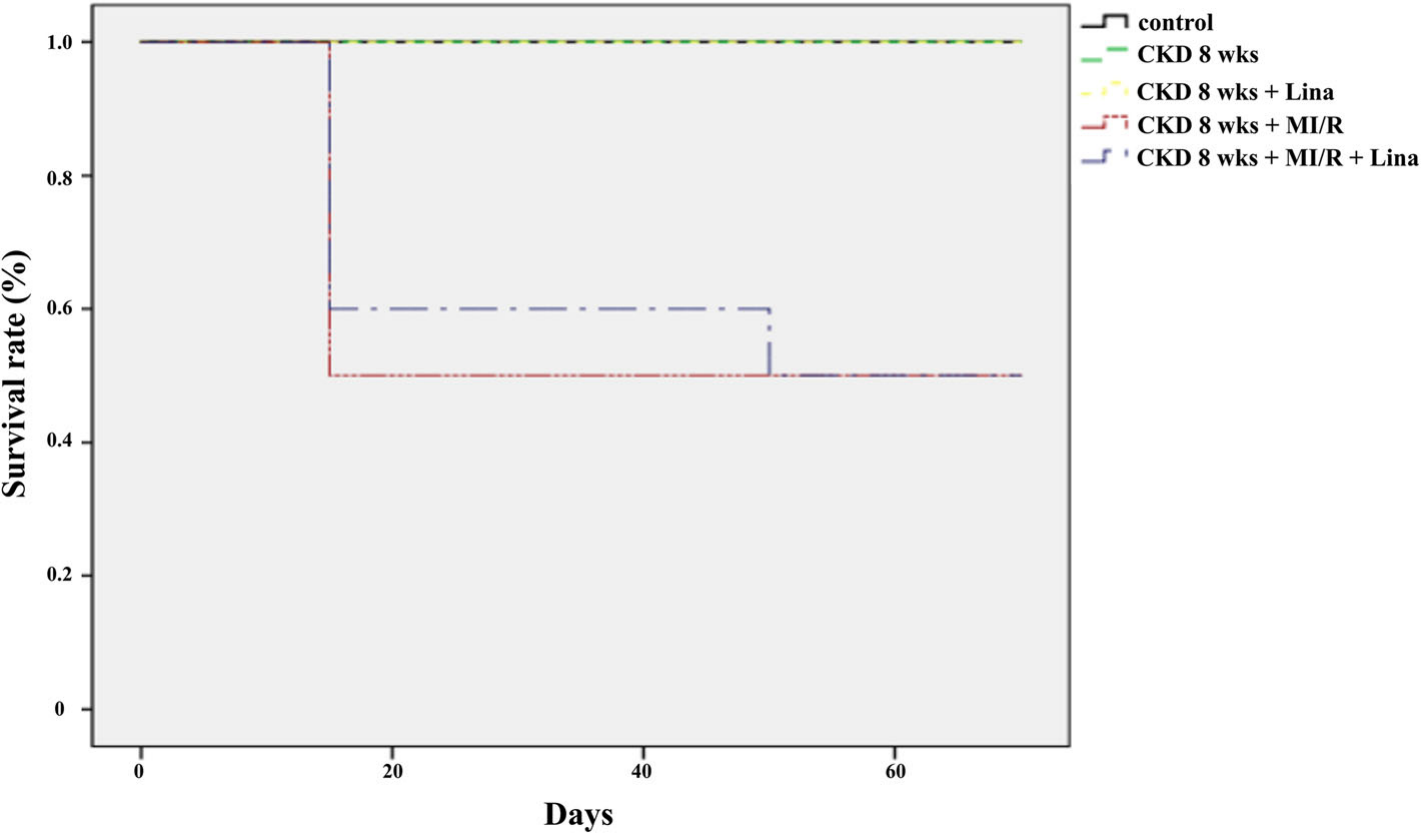
Kaplan–Meier plot for conditions of control, CKD, CKD-Li, CKD-MI/R and CKD-MI/R-Li associated with animal survival. CKD, chronic kidney disease; MI/R, myocardial ischemia/reperfusion; Li, linagliptin treatment

Seventy-day-survival of the different animal groups were 100% (control, CKD and CKD-Li), and 50% (CKD-MI/R, CKD-MI/R-Li) (Fig. 2). Serum creatinine and urine output were significantly increased during CKD progression compared with the controls 2–8 weeks after 5/6 nephrectomy (*p* < 0.01); blood pressure was also increased (*p* < 0.01). Animals with 5/6 nephrectomy also showed reduced body weight during CKD progression, relative to sham-operated controls (*p* < 0.05), with no difference between CKD and linagliptin-treated CKD. Serum albumin was also decreased after CKD progression (*p* < 0.05). Blood pressure was shown to be increased after CKD development (78.0 ± 9.8 mmHg in control vs. 137.4 ± 10.0 mmHg in CKD at 2 weeks, *p* < 0.05), but not during CKD progression (CKD, 2 vs. 4 weeks, *p* = 0.291; 4 vs. 8 weeks, *p* = 0.758). Urine pH was elevated after 4 weeks of CKD, relative to controls (*p* < 0.01); however, these changes were not evident in early CKD (control vs. CKD, 2 weeks, *p* = 0.492; Table 1).

**Table 1:** Clinical parameters of chronic kidney disease (CKD) rats

	Control	CKD 2 wks	CKD 4 wks	CKD 8 wks
Body Weight (g)	430.8 ± 18.4	319.4 ± 26.1^**^	334.5 ± 0.7^**^	371.3 ± 47.5^*^
24 Hr urine volume (mL)	14.8 ± 5.1	32.4 ± 10.0^**^	32.0 ± 0.0^**^	43.0 ± 12.3^**^
Urine pH	7.19 ± 0.50	7.38 ± 0.35	8.85 ± 0.04^** ††^	7.35 ± 0.13^‡‡^
Systolic blood pressure (mmHg)	78.0 ± 9.8	137.4 ± 10.0^**^	138.5 ± 10.0^**^	141.0 ± 7.5^**^
Glucose (mg/dL)	453.3 ± 56.9	277.5 ± 37.5^*^	299.5 ± 2.1^*^	298.3 ± 44.8^*^
Serum Cr (mg/dL)	0.28 ± 0.02	1.55 ± 0.09^**^	1.52 ± 0.06^**^	1.59 ± 0.10^**^
BUN (mg/dL)	19.3 ± 4.5	41.7 ± 7.8	43.3 ± 4.9^*^	42.3 ± 5.6^*^
Serum albumin (mg/dL)	2.1 ± 0.1	1.2 ± 0.1^*^	1.4 ± 0.1^*^	1.2 ± 0.1^**^
Cl_Cr_ (ml/min/100 g)	0.60 ± 0.06	0.07 ± 0.01^**^	0.08 ± 0.04^*^	0.09 ± 0.03^**^

*Cr* Creatinine, *BUN* Blood urea nitrogen, *Cl*_*Cr*_ Creatinine clearance, *Control* Sham-operated, CKD 2, 4, 8 weeks. Values are presented as the mean ± standard deviation (SD). n = number of rats. *p < 0.05 vs. control, **p < 0.01 vs. control

### Serum DPP-4 level was increased during progression of CKD

**Fig. 3:**
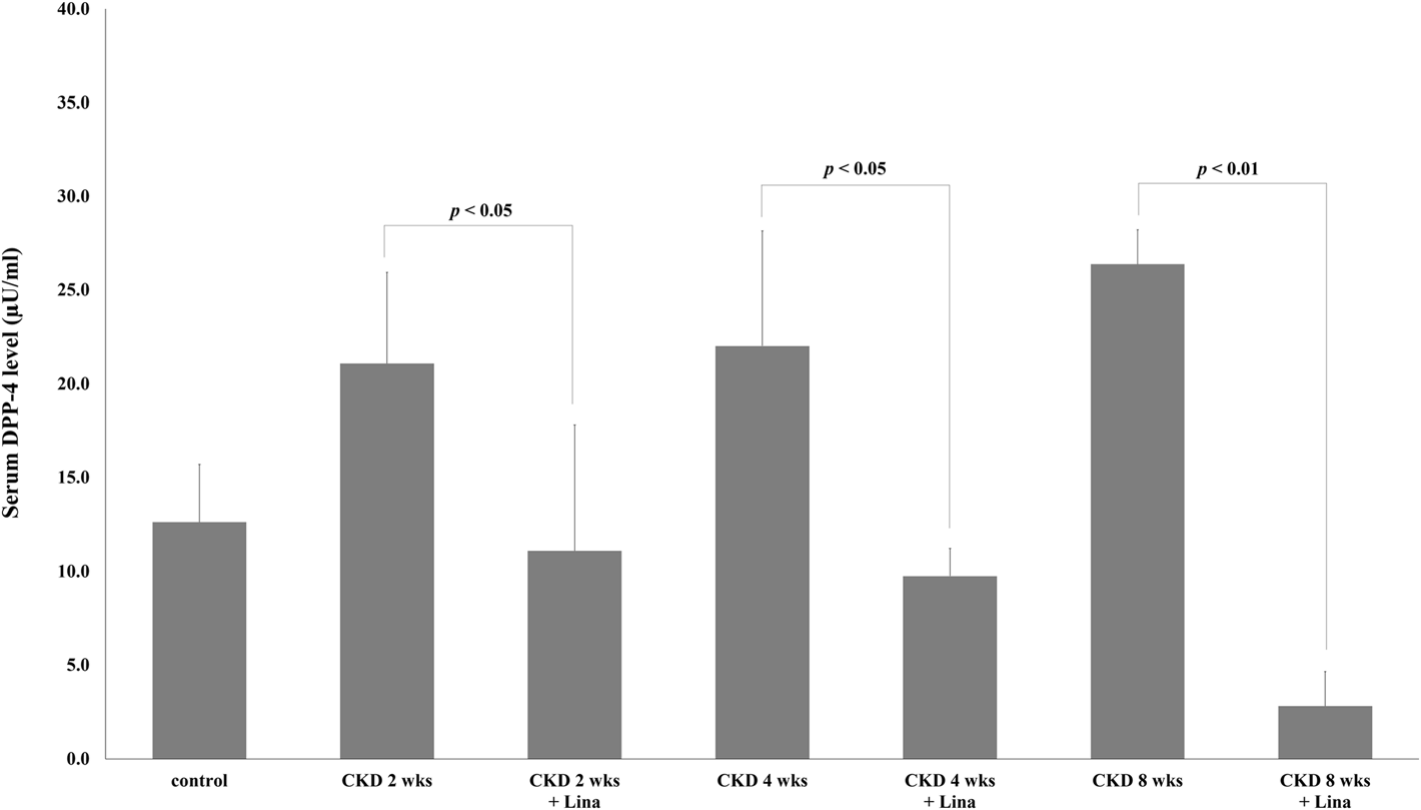
Dipeptidyl-peptidase 4 (DPP-4) activity in chronic kidney disease (CKD) and CKD with linagliptin-treated rats. Control, sham-operated; CKD 2, 4, 8 weeks, 5/6 nephrectomized rats 2, 4, and 8 weeks post-treatment; Lina, linagliptin. Values are presented as the mean ± standard deviation (SD)

Serum DPP-4 level was increased in the CKD group, relative to control (2 weeks, 21.1 ± 4.8; 4 weeks, 22.0 ± 6.1; and 8 weeks, 26.4 ± 1.8; vs. 12.6 ± 3.1 for controls; *p* < 0.05). Although DPP-4 levels were highest in 8-week CKD animals, these differences were not statistically significant compared with earlier time points (CKD, 2 vs. 4 weeks, *p* = 0.808; CKD, 4 vs. 8 weeks, *p* = 0.226). Serum DPP-4 levels were markedly decreased following linagliptin treatment, with the largest differences seen at 8 weeks (26.4 ± 1.8 vs. 2.8 ± 1.8 for CKD and CKD-Lina 8 weeks, respectively; *p* < 0.01; Fig. 3). Differences between earlier time points were not statistically significant (2 vs. 4 weeks, *p* = 0.34).

### Linagliptin ameliorated kidney injury in the CKD rat

**Fig. 4:**
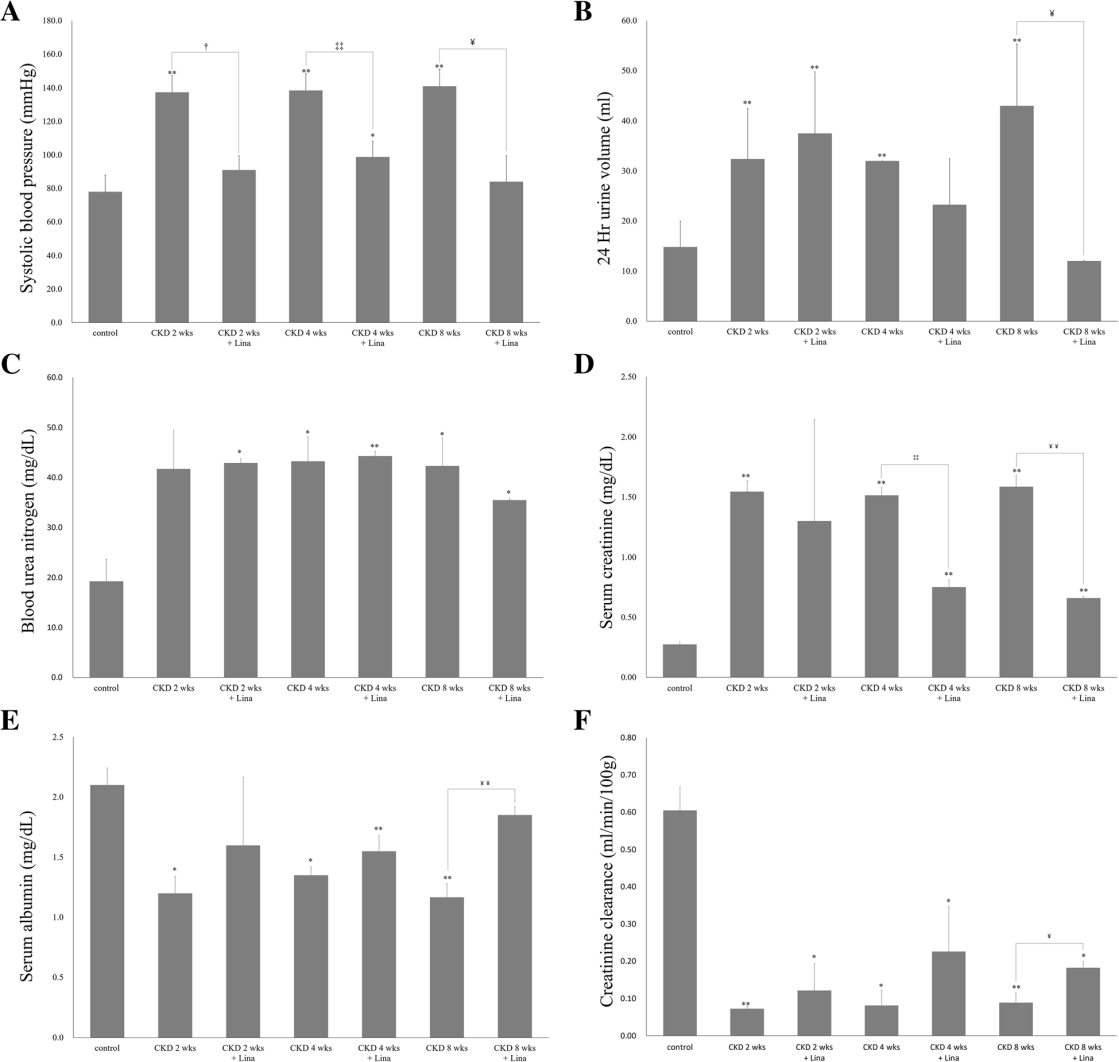
Change in treatment outcomes in CKD and CKD with linagliptin-treated rats. Blood pressure (**a**), urine volume (**b**), blood urea nitrogen (**c**), serum creatinine (**d**), albumin (**e**), and creatinine clearance (**f**). Control, sham-operated; CKD 2, 4, and 8 weeks, 5/6 nephrectomized rats 2, 4, and 8 weeks post-treatment; Lina, linagliptin. Values are presented as the mean ± SD. *p < 0.05 vs. control; **p < 0.01 vs. control; ^†^*p* < 0.05 vs. CKD, 2 weeks; ^††^*p* < 0.01 vs. CKD, 2 weeks; ^‡^*p* < 0.05 vs. CKD, 4 weeks; ^‡‡^*p* < 0.01 vs. CKD, 4 weeks; ^¥^*p* < 0.05 vs. CKD, 8 weeks; and ^¥¥^*p* < 0.01 vs. CKD, 8 weeks

Systolic blood pressure was increased after CKD development, though these changes were not evident during CKD progression. In contrast, blood pressure was significantly decreased at all three CKD time points after linagliptin treatment (*p* < 0.05; Fig. 4a). The volume of 24 h urine was markedly increased 2 weeks after 5/6 nephrectomy, with no differences evident between the 2 and 4 weeks points for either CKD or CKD with linagliptin treatment (CKD, 2 weeks vs. CKD-Lina, 2 weeks, *p* = 0.513; CKD, 4 weeks vs. CKD-Lina, 4 weeks, *p* = 0.274). However, this trend was not evident at later time points, with urine output significantly decreased in the CKD-Lina group 8 weeks after surgery (43.0 ± 12.3 in CKD, 8 weeks vs. 12.1 ± 0.1 mL/day in CKD-Lina, 8 weeks, *p* < 0.05; Fig. 4b). Although serum urea nitrogen levels exhibited no change after treatment (Fig. 4c), serum creatinine was significantly decreased both 4 and 8 weeks after surgery in linagliptin-treated animals relative to the CKD group (1.52 ± 0.06 in CKD, 4 weeks vs. 0.75 ± 0.06 in CKD-Lina, 4 weeks, *p* < 0.01; 1.59 ± 0.10 in CKD, 8 weeks vs. 0.66 ± 0.01 in CKD-Lina, 8 weeks, *p* < 0.01; Fig. 4d). Improvements in creatinine clearance were evident by 8 weeks of 5/6 nephrectomy (Fig. 4f). Serum albumin levels were also increased in the CKD-Lina group (*p* < 0.05; Fig. 4e).

### Renal tubular GLP-1R expression in the CKD rat

**Fig. 5:**
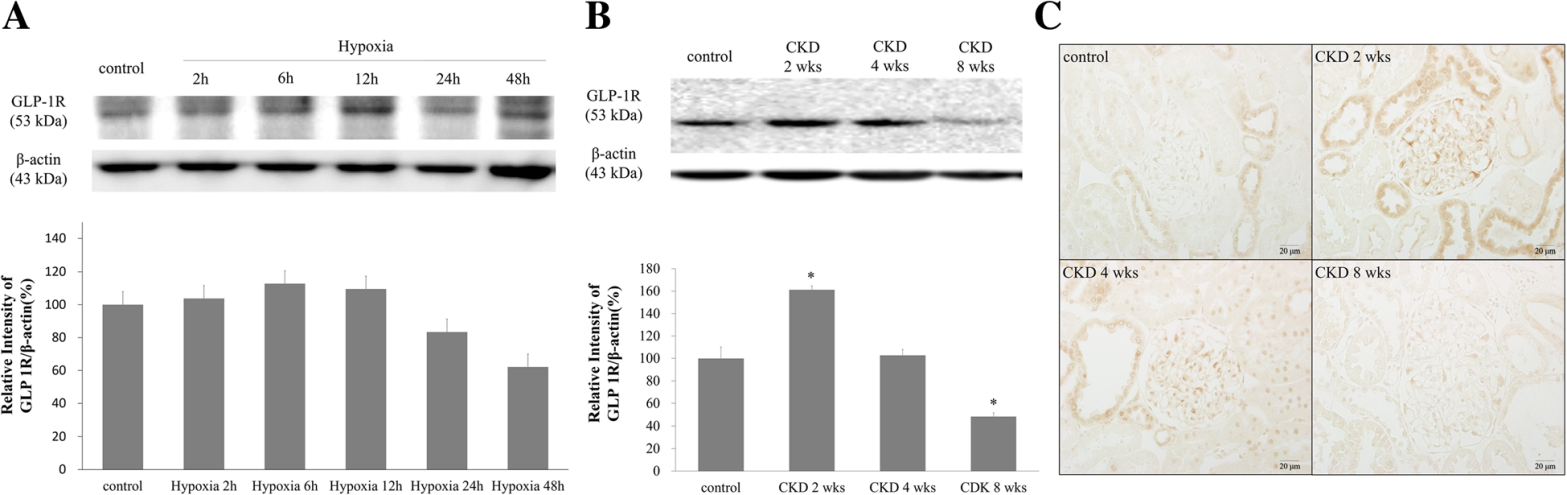
Glucagon-like peptide-1 receptor (GLP-1R) expression in the CKD rat. **a** Western blot analysis of GLP-1R expression in rat kidney proximal tubule (NRK-52E) cells with hypoxic injury. **b** Western blot analysis of renal cortical GLP-1R expression in 5/6 nephrectomized rat. **c** Renal cortical GLP-1R immunoreactivity in CKD rats. Control, sham-operated; CKD 2, 4, 8 weeks, 5/6 nephrectomized rats 2, 4, and 8 weeks post-treatment. Black lines, separated from adjacent bands. Values are presented as the mean ± SD. *p < 0.05 vs. control. Magnification 400 ×. * The original blots of fig 5-11 are in Additional file 1: Figure S1.

Under hypoxic conditions, NRK-52E cellular GLP-1R expression was the highest 6 h after initiation of hypoxia, with decreases in expression thereafter. By 48 h post-hypoxia, GLP-1R levels had risen to 62% of the control (Fig. 5a). The density of the renal cortical GLP-1R band on WB was markedly increased 2 weeks after 5/6 nephrectomy; however, it decreased to control levels 4 weeks after surgery. Eight weeks after CKD onset, GLP-1R expression was 61% of that of the control group (Fig. 5b). IHC showed similar results to those of WB (Fig. 5c). The peak intensity of GLP-1R expression was noted 2 weeks post-CKD onset; expression started to decrease after 4 weeks and decreased markedly 8 weeks after surgery.

**Fig. 6:**
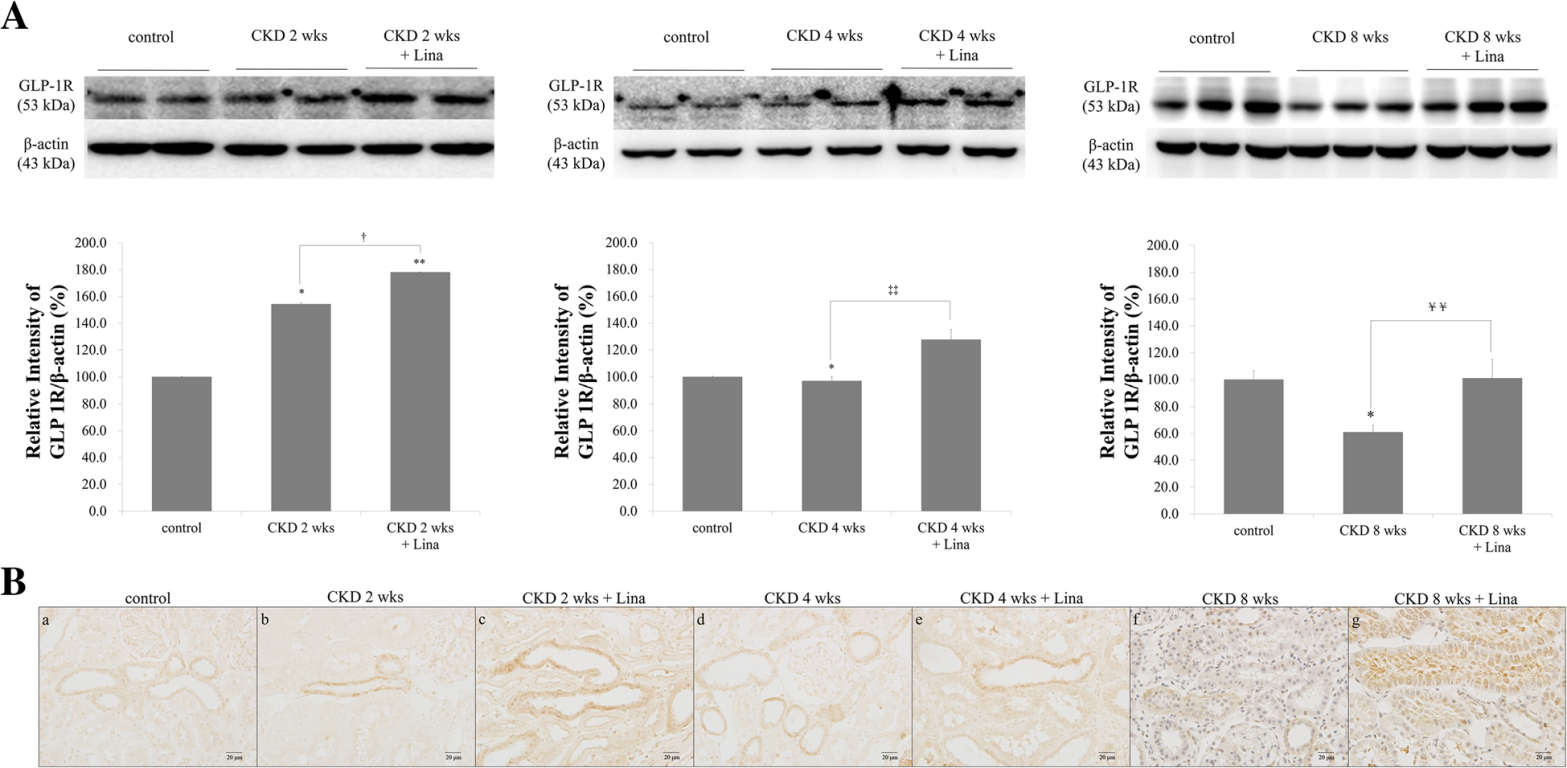
Effect of linagliptin in CKD and CKD with linagliptin-treated rats. **A** Western blot analysis of CKD and CKD with linagliptin-treated rats. **B** GLP-1R immunoreactivity in the renal cortical proximal tubule of CKD and CKD with linagliptin-treated rats. a, control; b, CKD, 2 wks; c, CKD, 2 weeks with linagliptin-treated; d, CKD, 4 wks; e, CKD, 4 weeks with linagliptin-treated; f, CKD, 8 wks; g, CKD, 8 weeks with linagliptin-treated; Values are presented as the mean ± SD. *p < 0.05 vs. control; **p < 0.01 vs. control; ^†^p < 0.05 vs. CKD, 2 weeks; ^††^*p* < 0.01 vs. CKD, 2 weeks; ^‡^*p* < 0.05 vs. CKD, 4 weeks; ^‡‡^*p* < 0.01 vs. CKD, 4 weeks; ^¥^*p* < 0.05 vs. CKD 8, weeks; and ^¥¥^*p* < 0.01 vs. CKD, 8 weeks. Magnification 400 ×

Linagliptin treatment enhanced GLP-1R expression in the renal cortex. GLP-1R was increased 2 weeks after CKD onset in the linagliptin treatment group, with incremental increases in GLP-1R density on WB evident at 4 and 8 weeks (Fig. 5b). Similar results were seen with IHC, with staining intensity increasing incrementally after linagliptin treatment during each stage of CKD (2 weeks, 154.4 ± 3.0 vs. 178.1 ± 0.1; 4 weeks, 97.0 ± 0.4 vs. 127.8 ± 7.4; 8 weeks, 61.0 ± 5.0 vs. 101 ± 13.8, without and with treatment, respectively; *p* < 0.05, relative to control; Fig. 6A). Linagliptin treatment significantly enhanced tubular GLP-1R expression, with higher levels of GLP-1R staining evident in the proximal tubular area (Fig. 6B), which recovered to control levels even at 8 weeks after CKD onset.

### Renal tubular GLP-1R change in CKD-MI

**Fig. 7:**
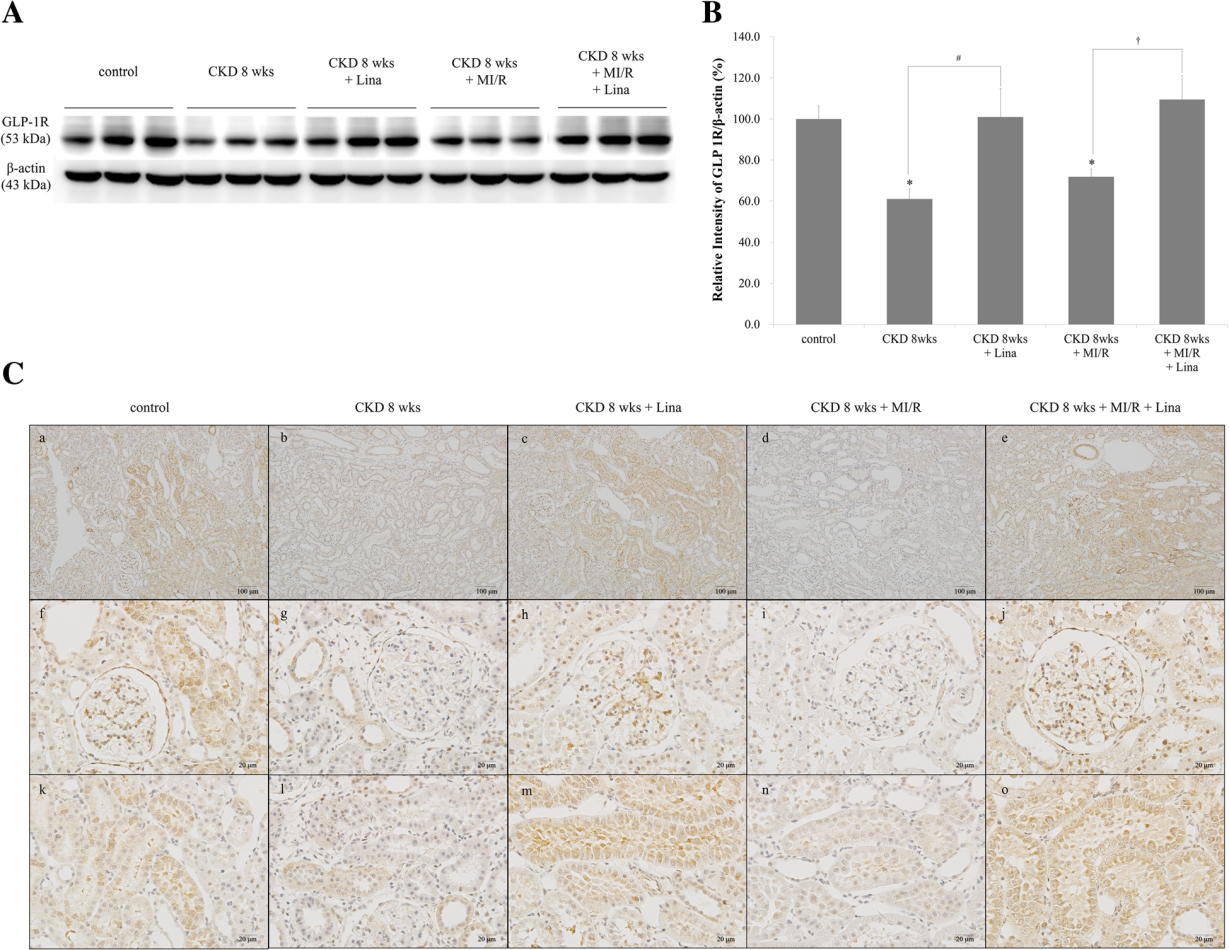
Expression of renal tubular GLP-1R. **A** Western blots showing GLP-1R protein levels in the renal cortex of control; CKD, 8 weeks; CKD, 8 weeks + Lina; CKD, 8 weeks + MI/R; and CKD, 8 weeks + MI/R + Lina rats. **B** Quantification of GLP-1R protein levels were standardized based on β-actin expression in the renal cortex. **C** GLP-1R immunoreactivity of the renal cortex. a, f, and k, control; b, g, and i, CKD, 8 weeks; c, h, and m, CKD, 8 weeks with linagliptin-treated; d, i, and n, CKD, 8 weeks and MI/R; e, j, and o, CKD, 8 weeks and MI/R with linagliptin-treated group. CKD, chronic kidney disease; MI/R, myocardial ischemia/reperfusion; Lina, linagliptin. Black lines, separated from adjacent bands. Values are presented as the mean ± SD. *p < 0.05 vs. control. a–e, magnification 100×; f–o, magnification 400 ×

WB revealed significant decreases in renal tubular GLP-1R expression 8 weeks after CKD and CKD with myocardial ischemia. GLP-1R protein levels were significantly increased in both CKD and CKD with myocardial ischemia groups after linagliptin treatment (Fig. 7A-B). IHC analyses revealed similar results as WB, with increased GLP-1R expression in the renal cortical tubule after linagliptin treatment (Fig. 7C).

### Myocardial GLP-1R expression in acute stage of ischemia

**Fig. 8:**
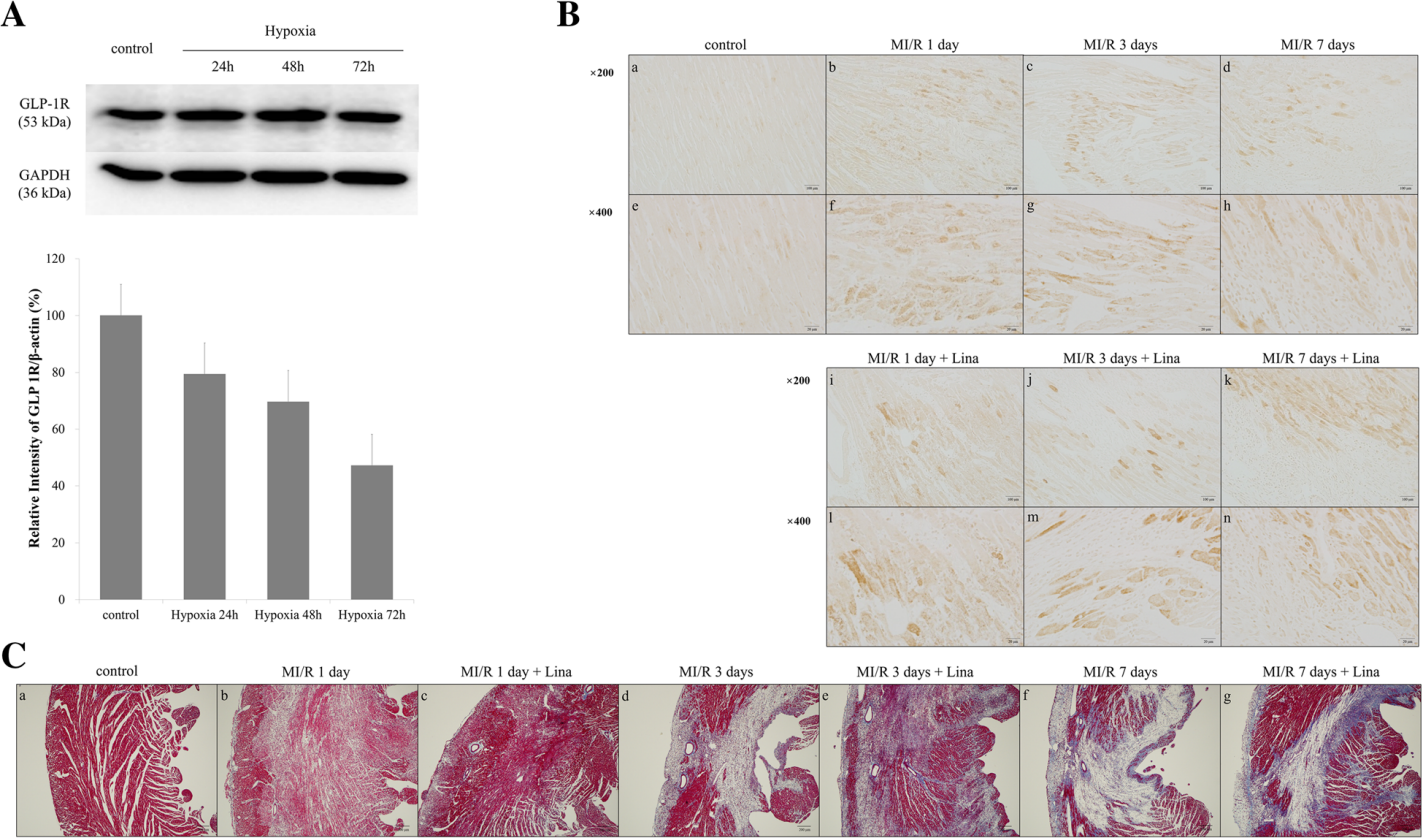
GLP-1R in cardiac progenitor cells and myocardium by ischemia. **A** Western blot of cardiac progenitor cells with hypoxic injury. **B** GLP-1R Immunoreactivity of myocardium. a and e, control; b and f, MI/R 1 day; c and g, MI/R 1 days; d and h, MI/R 7 days; i and l, MI/R 1 day with linagliptin-treated; j and m, MI/R 3 day with linagliptin-treated; k and n, MI/R 7 day with linagliptin-treated group. **C** Masson’s Trichrome staning of myocardium. a, control; b, MI/R 1 day; c, MI/R 1 day with linagliptin-treated; d, MI/R 3 days; e, MI/R 3 day with linagliptin-treated; f, MI/R 7 day; g, MI/R 7 day with linagliptin-treated group. MI/R, myocardial ischemia/reperfusion; Lina, linagliptin. **A** a-d and i-k, magnification 200×; e-h and l-m, magnification 400×. **C** a-g, magnification 40 ×

Cellular GLP-1R expression was decreased after acute ischemic damage to CMCs. After ischemic injury, WB analysis of CMCs revealed gradual decreases in GLP-1R over 72 h, though these changes were not statistically significant (Fig. 8A). IHC analysis of myocardial tissues showed increased GLP-1R in the ischemic area after LAD ligation, relative to control, though differences between days 1, 3, and 7 post-myocardial ischemia were not statistically significant (Fig. 8B). Ischemic myocardium showed significant wall thinning and fibrotic change by Masson’s trichrome stain 3 and 7 days after ischemia, which explained myocardial remodeling (Fig. 8C; d and f). Interestingly, this fibrotic area and wall thinning were markedly attenuated after linagliptin treatment (Fig. 8C; e and g).

### Myocardial GLP-1R changes after DPP-4 inhibition in CKD-MI

**Fig. 9:**
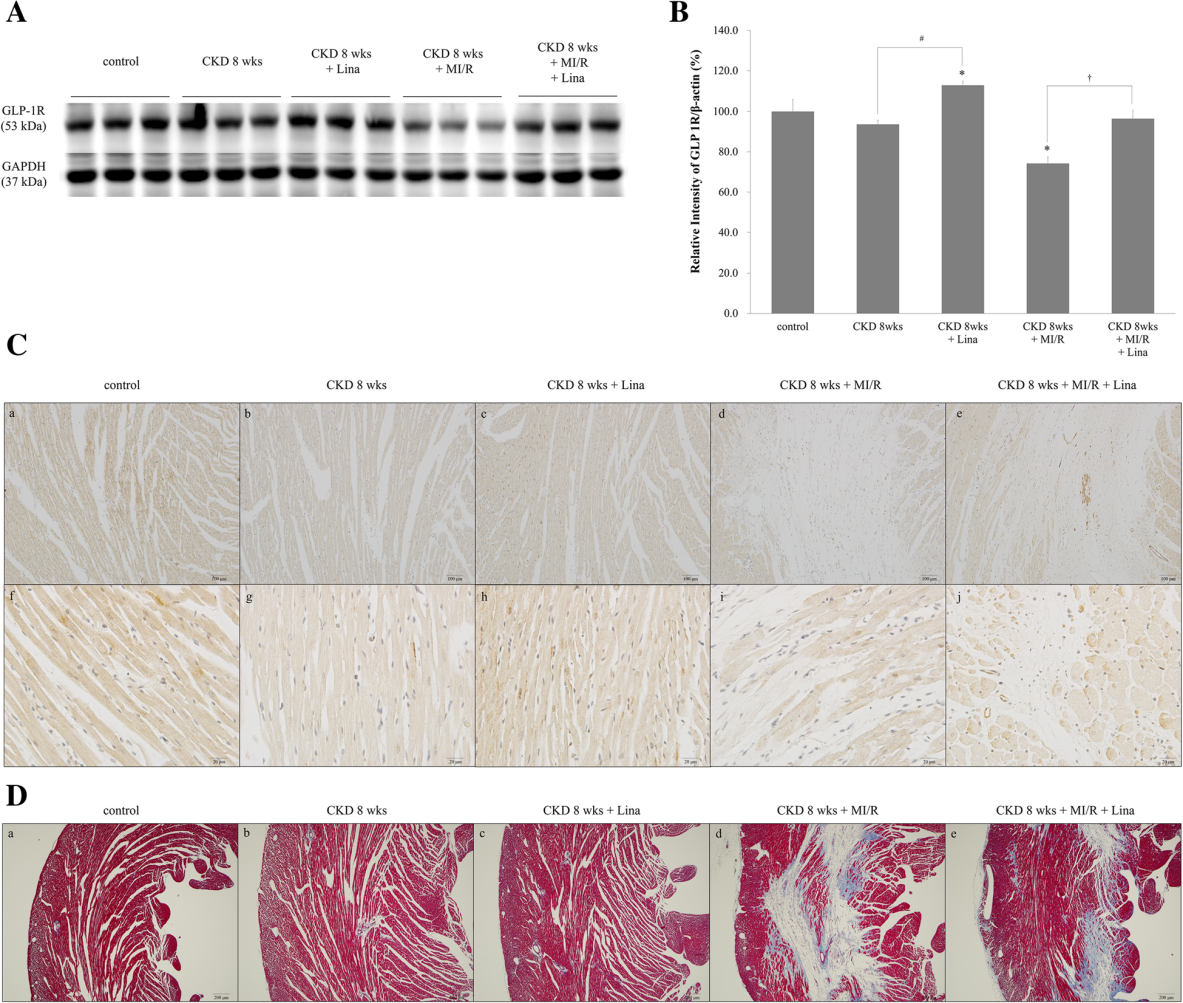
Expression of myocardial GLP-1R. **A** Western blot analysis of protein levels in the myocardium of control; CKD, 8 weeks; CKD, 8 weeks + Lina; CKD, 8 weeks + MI/R; and CKD, 8 weeks + MI/R + Lina rats. **B** Quantification of GLP-1R protein levels was standardized based on glyceraldehyde 3-phosphate dehydrogenase (GAPDH) expression in the myocardium. **C** GLP-1R immunoreactivity of myocardium. a and f, control; b and g, CKD, 8 weeks; c and h, CKD, 8 weeks with linagliptin-treated; d and i, CKD, 8 weeks and MI/R; e and j, CKD, 8 weeks and MI/R with linagliptin-treated group. **D** Masson’s Trichrome staning of myocardium. a, control; b, CKD, 8 weeks; c, CKD, 8 weeks with linagliptin-treated; d, CKD, 8 weeks and MI/R; e, CKD, 8 weeks and MI/R with linagliptin-treated group. CKD, chronic kidney disease; MI/R, myocardial ischemia/reperfusion; Lina, linagliptin. Values are presented as the mean ± SD. *p < 0.05 vs. control. **B** a–e, magnification 100×; f–o, magnification 400×, **C** a–e, magnification 100 ×

WB of myocardial GLP-1R was significantly increased 8 weeks after CKD onset, with the strongest changes seen in CKD with myocardial ischemia. After linagliptin treatment, the WB density of GLP-1R was significantly increased in both the CKD and CKD with myocardial ischemia groups (Fig. 9A and B). IHC showed similar results as WB, with increased expression evident in the border of the ischemic area of the myocardium (Fig. 9C). Masson’s trichrome stain showed histological change increased ischemic area in myocardium by LAD ligation. Ischemic area was decreased by linagliptin treatment compared with CKD-MI/R group (Fig. 9D).

### Renal cortical phosphorylation and expression of ERK1/2 and Bcl-2 in CKD and CKD with MI/R

**Fig. 10:**
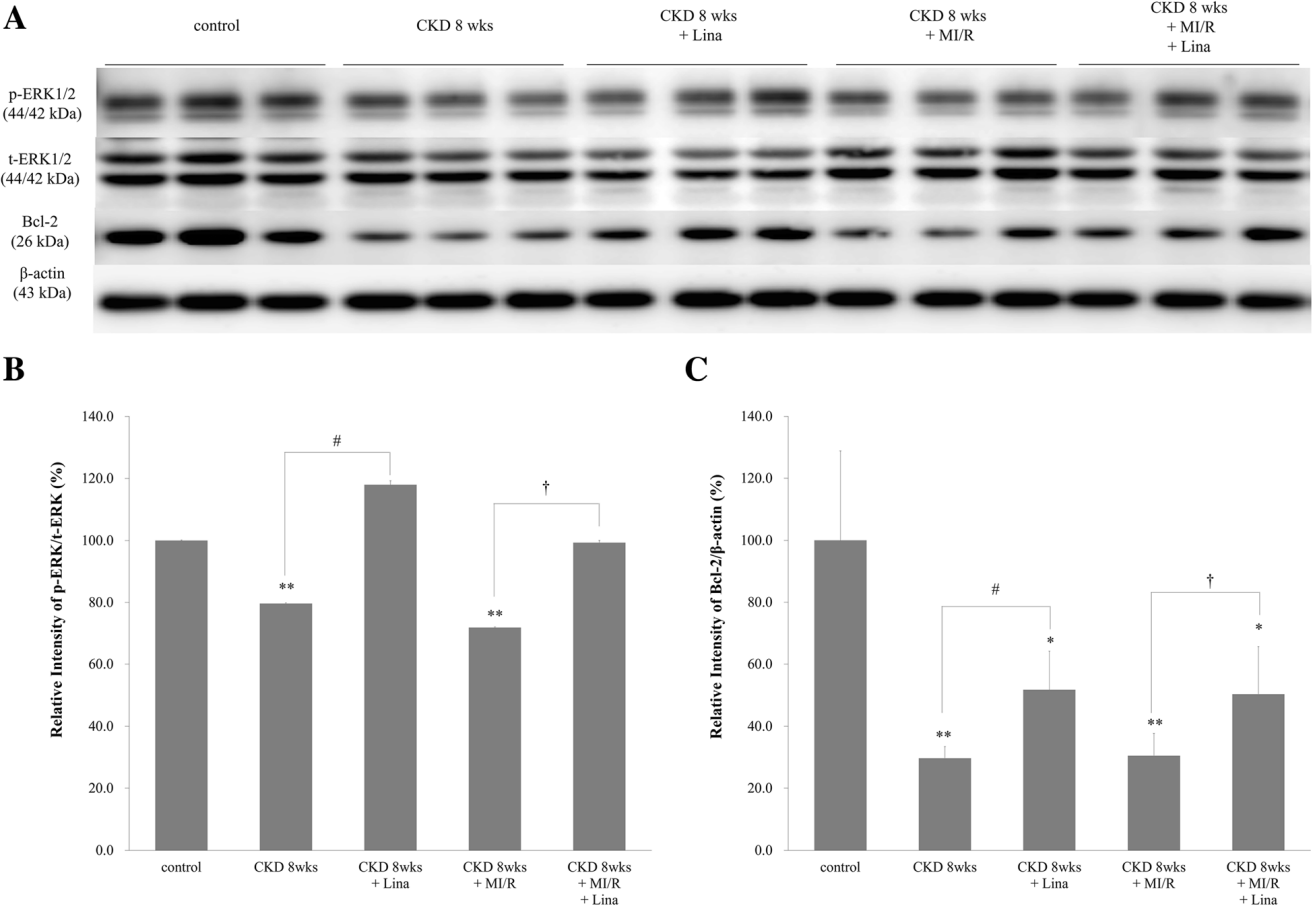
Phosphorylation of extracellular signal-regulated kinase (EKR1/2) and expression of B cell lymphoma-2 (Bcl-2) in the renal cortex. **a** Western blot showing protein levels in renal cortex from control; CKD, 8 weeks; CKD, 8 weeks + Lina; CKD, 8 weeks + MI/R; CKD, 8 weeks + MI/R + Lina. **b** and **c** Quantification of p-ERK1/2 and Bcl-2 protein levels was standardized relative to t-ERK1/2, and β-actin expression in the renal cortex. Values are presented as the mean ± SD. *p < 0.05 vs. control; ^**^p < 0.01 vs. control; ^#^*p* < 0.05 vs. CKD, 8 weeks; ^†^*p* < 0.05 vs. CKD, 8 weeks + MI/R

Renal cortical expression of Bcl-2 and phosphorylated ERK1/2 (p-ERK1/2) were all decreased in the CKD and CKD with MI/R rat, with these decreases markedly attenuated following linagliptin treatment (Fig. 10a-c).

### Myocardial ERK1/2 and Bcl-2 expression in CKD and CKD with MI/R

**Fig. 11:**
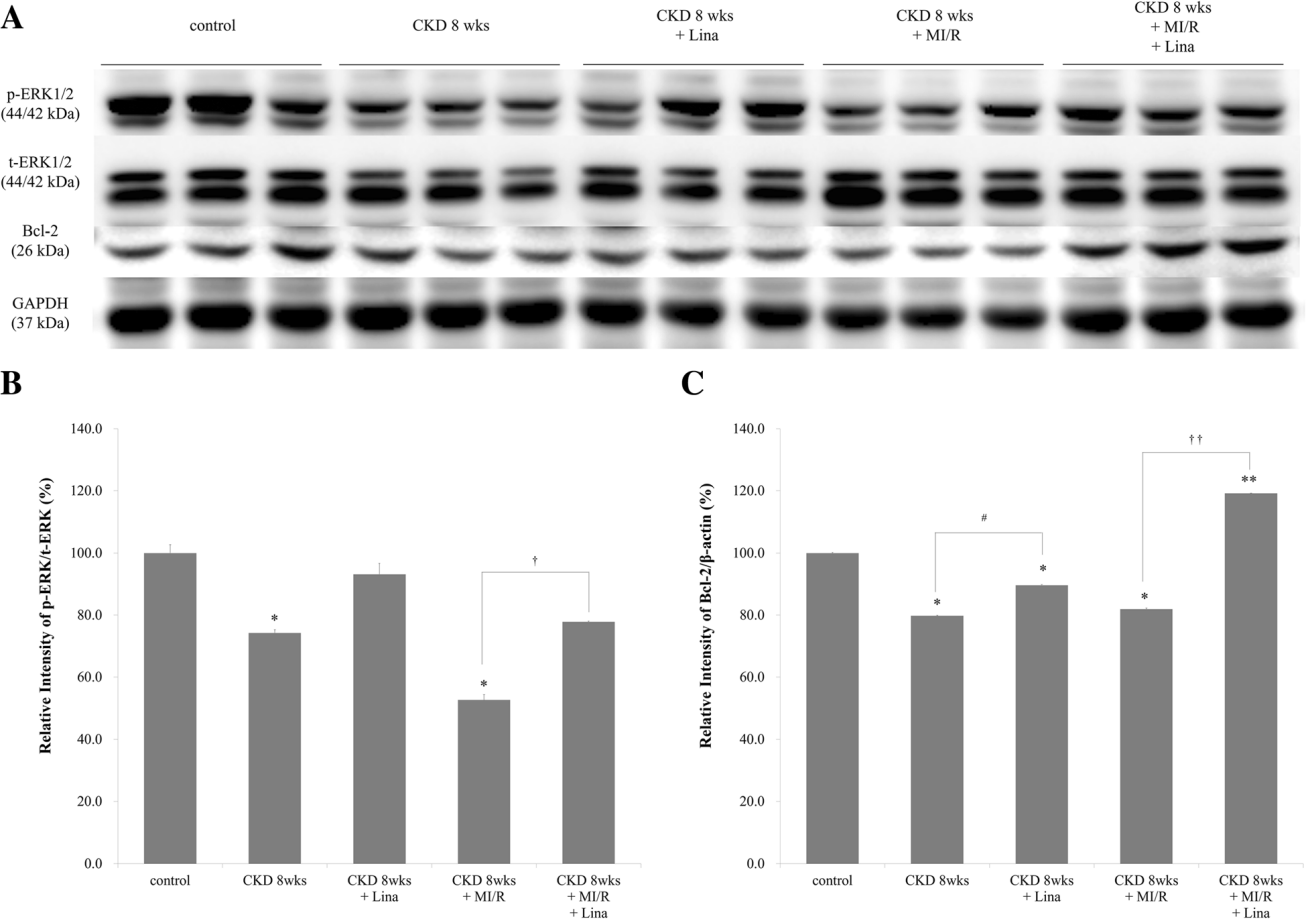
Phosphorylation of EKR1/2 and expression of Bcl-2 in the myocardium. **a** Western blot showing protein levels in control, CKD with/without linagliptin, and CKD-MI with/without linagliptin rats. **b** and **c** Quantitation of p-ERK1/2 and Bcl-2 standardized relative to t-ERK1/2, and GAPDH expression in the renal cortex. *p < 0.05 vs. control; ^#^*p* < 0.05 vs. CKD, 8 weeks; ^†^*p* < 0.05 vs. CKD, 8 weeks + MI/R; ^††^*p* < 0.01 vs. CKD, 8 weeks + MI/R

Myocardial p-ERK1/2 and Bcl-2 expression were compared among control, CKD, CKD-Lina, and CKD with MI/R with and without linagliptin treatment. All groups exhibited decreased protein expression after 5/6 nephrectomy, in combination with increased expression after linagliptin treatment (Fig. 11a). The largest difference was seen in the CKD with MI/R group (Fig. 11b–c).

## Discussion

We investigated renal tubular and myocardial GLP-1R expression in CKD with MI/R before and after DPP-4 inhibition. Although we did not measure serum GLP-1 level in the experiment, decreased DPP-4 level, typically measured in the plasma, is another potential mechanism that GLP-1 may also have beneficial effects in the setting of kidney and cardiovascular disease. GLP-1 is an incretin hormone that stimulates insulin secretion and forms the basis of a drug class for type 2 diabetes treatment. However, half-life of native GLP-1 is within 2 min in vivo, which is due to degradation by the DPP-4 [18], it is hard to define rapid change after DPP-4 inhibition. Also, human data already show levels of GLP-1 protein after DPP-4 inhibition [19], and many other articles proved its change [20–25]. Despite evidence presented here, the relationship between DPP-4 activity and CKD remains unclear. Although Joo et al. reported no changes in DPP-4 activity at 8 weeks of CKD [26], we found the DPP-4 inhibitory activity of linagliptin to be much higher in advanced CKD. DPP-4 activity was highest 8 weeks post-CKD onset, with the biggest changes after linagliptin treatment also observed at this time point. As linagliptin passes mainly through the enterohepatic circulation, its potent effect in CKD may be associated with not only higher blood levels but also a higher binding affinity to DPP-4.

We found GLP-1R expression was increased after DPP-4 inhibition, and it is thought to be associated with ligand-receptor interaction. It is well established, in cellular signaling fields, that the level of various cellular receptors is proportionally related to the extracellular level of their cognate ligands [27–29]. Linagliptin is reported to increase the level of GLP-1 and under this condition, is reasoned to increase the level of GLP-1 receptor and its downstream signaling activity. Indeed, we demonstrate that Linagliptin increases not only GLP-1R but also its known downstream signaling to PKA, AKT and ERK, probably through up-regulation of GLP-1. GLP-1 exerts its biological actions via binding to GLP-1R [30] on the surface of various cells including pancreas, heart and kidney [11, 12]. Also, the GLP-1R is a G-protein coupled receptor that stimulates adenylate cyclase and PKA. Kuna RS et al. demonstrated that GLP-1R/ligand complex couples to adenylyl cyclase in endosome [31].

We also found that DPP-4 activity increased in conjunction with the progression of CKD. Previous studies have also shown decreases in DPP-4 activity after DPP-4 inhibitor treatment [26, 32], but none have demonstrated higher DDP-4 activity in advanced CKD. If these effects are mediated by increased production, the kidneys may play an important role in DPP-4 activity. If caused by decreased renal clearance, the increase in GLP-1 inhibition associated with DPP-4 activity may aggravate vascular complications in CKD. Further studies investigating DPP-4 inhibition in CKD are warranted to determine this association.

DPP-4 inhibition has been shown to prevent cardiovascular complication and ameliorate kidney injury in previous studies [33–35]. Rats with CKD showed decreased body weight and serum albumin, combined with increased urine output and serum creatinine. Similar results have also been observed in humans [20]. Here, we found that linagliptin treatment prevented CKD-mediated increases in blood pressure and serum creatinine. The rats in this study were not diabetic, and even with high-dose linagliptin, analysis of serum glucose levels showed no hypoglycemia in the linagliptin treatment group. The linagliptin treated group also exhibited significant decreases in urine output, relative to untreated CKD rats, which may be indicative of preserved tubular urine concentration ability. Future studies will be necessary to confirm such an effect.

GLP-1R is thought to be expressed in the glomeruli and proximal tubule [36], with increased expression evident in an ischemic reperfusion model of acute kidney injury [37]. Here, we definitively show by WB and IHC that GLP-1R is expressed at the proximal tubule of the renal cortex, with increased expression evident in the early stage of CKD, followed by decreases thereafter. This analysis of renal GLP-1 activity in CKD is important due to the changes in expression observed over time. GLP-1R expression was markedly increased 2 weeks after 5/6 nephrectomy, followed by a decrease to control levels at 4 weeks, with further decreases to 61% of control levels 8 weeks after CKD onset. While Joo et al. had also reported that renal GLP-1R levels were decreased 8 weeks after 5/6 nephrectomy [26]; to the best of our knowledge, no reports have demonstrated an elevation in GLP-1R levels in early CKD, nor demonstrated alterations in gene expression during CKD progression.

As glomerulosclerosis develops by 4 weeks in CKD models and tubulointerstitial fibrosis develops by 8 weeks [38], we estimate that functioning tubules increase their expression of GLP-1R over the first 2 weeks, followed by decreased expression by week 8 as a result of tubular fibrosis and apoptosis. If GLP-1 is involved in vascular protection, early increases in GLP-1R levels may signify a protective function that occurs during stressful conditions, such as tubular ischemia, which arises during the early stages of CKD. Similar to other studies, we also found that renal cortical GLP-1R expression was increased by DPP-4 inhibition [26, 37], and that these changes were biggest even in instances of low GLP-1R expression. Taken together, these data suggest that increased GLP-1R expression may be associated with renoprotection in CKD.

DPP-4 inhibitors have been shown to confer protection against ischemic injury of the myocardium [39–41], as well as prevent progression of uremic cardiomyopathy in a rat model of CKD [21]. Here, we also showed decreased GLP-1R expression in the ischemic myocardium of the CKD rat, which was restored following linagliptin treatment.

Next, we compared cellular and in vivo changes in GLP-1R expression in the myocardium. In acute ischemia, GLP-1R was decreased in myocardial progenitor cells (Fig. 9A) but increased in myocardium in vivo (Fig. 9B). Myocardial increment of GLP-1R during acute ischemia was similar to that seen in the renal tubule; however, changes in progenitor cells were different. The limitation of this our study was not performed echocardiography for functional readouts of heart function. However, we described myocardial remodeling (thinning) and myocardial fibrosis in the manuscript like other researches [42, 43]. Further studies will be necessary to identify cellular interactions within the myocardium in the ischemic milieu. Previous studies have shown conflicting outcomes regarding the role of myocardial ischemia in CKD, from those showing no association with renal disease progression [44] to those reporting exacerbation of kidney injury [45, 46]. The data presented here may serve as an important reference regarding the role of ischemic heart disease in CKD. As DPP-4 activity is highest in the kidney [47], increased DPP-4 activity in CKD may worsen myocardial ischemia.

Recent studies indicate that apoptosis plays an important role in both acute and chronic CKD, as well as myocardial ischemia. Apoptosis accelerated tissue damage by stimulating tubular epithelial cell loss in acute and chronic kidney injury [48–50] as well as in myocardial ischemia and reperfusion injuries [51, 52]. ERK1/2, an important subfamily of MAPKs, regulate cellular transcriptional activities, cell proliferation, differentiation, and cell survival [53, 54]. The ERK1/2 cascade has been linked to ischemic preconditioning [55, 56] and plays an important role in myocardial protection against ischemia-reperfusion injury. Anti-apoptotic protein, Bcl-2 has been extensively studied in the kidney and heart. Renal tubular expression of Bcl-2 is decreased by severe cellular stimuli, and in acute kidney injury in vivo [57]. In our WB analyses, renal cortical and myocardial ERK1/2 phosphorylation and Bcl-2 expression were all increased in CKD with cardiac ischemia-reperfusion after linagliptin treatment (Figs. 10 and 11). As phosphorylation of ERK1/2 and activation of Bcl-2 are important pathways in CKD and MI/R injuries, our data indicate that linagliptin protects the myocardium and renal tubules from CKD with cardiac ischemia-reperfusion induced apoptosis via modulation of the ERK1/2 and Bcl-2 signaling pathways.

As with all research, this study has certain limitations. First, the DDP-4 dosages used in this study are higher than that of standard pharmacological dosages. However, the concentrations used did not cause hypoglycemia or cellular injury. Second, we did not compare myocardial ischemia without CKD. Third, we did not experiment by blinding test each group of allocation. Because of used surgical disease models (5/6th nephrectomy, myocardial ischemia reperfusion, 5/6th nephrectomy with/without myocardial ischemia reperfusion), it is hard to do blind experiment. However, to minimize the bias, we applied duplicate assessment of outcomes. Further investigations measuring changes in myocardial GLP-1R or GLP-1 activity in myocardial ischemia with and without CKD progression will be useful to better understand the cardio-protective effects of GLP-1 in CKD.

## Conclusions

Taken together, the data presented here show important, time-dependent effects of GLP-1R and DPP-4 inhibition during CKD progression. Renal tubular GLP-1R is increased in early CKD followed by decreased expression thereafter. Similarly, myocardial GLP-1R is also increased in early ischemia and decreased in later disease. DPP-4 inhibition is higher in advanced CKD, with linagliptin enhancing renal tubular and myocardial expression of GLP-1R. These data suggest that GLP-1R may confer important renal and cardio-protective effects in CKD, with DPP-4 inhibition exerting clear benefits, regardless of its hypoglycemic effects.

## Additional file

Additional file 1: **Figure S1.** Original blot of protein expression. Western blot bands including markers were imaged by a LAS-3000 imaging system (Fujifilm life science, Minato-ku, Tokyo). (TIF 2187 kb)

## Abbreviations

Bcl-2: B cell lymphoma-2
CKD: Chronic kidney disease
CKD-Lina: CKD with DPP-4 inhibitor
CKD-MI: CKD with myocardial ischemia/reperfusion
CKD-MI-Lina: CKD-MI with DPP-4 inhibitor
DPP-4: Dipeptidyl-peptidase 4
ERK1/2: Extracellular signal-regulated kinase
GLP-1: Glucagon-like peptide-1
GLP-1R: GLP-1 receptor
IHC: Immunohistochemistry
LAD: Left anterior descending branch of coronary artery
MI/R: Myocardial ischemia/reperfusion
WB: Western blot
