# Supplementary figures and images for: DPP-4 inhibition enhanced renal tubular and myocardial GLP-1 receptor expression decreased in CKD with myocardial infarction
Source: BMC Nephrol. 2019 Mar 1;20:75. doi: 10.1186/s12882-019-1243-z (PMC6397488; doi:10.1186/s12882-019-1243-z)

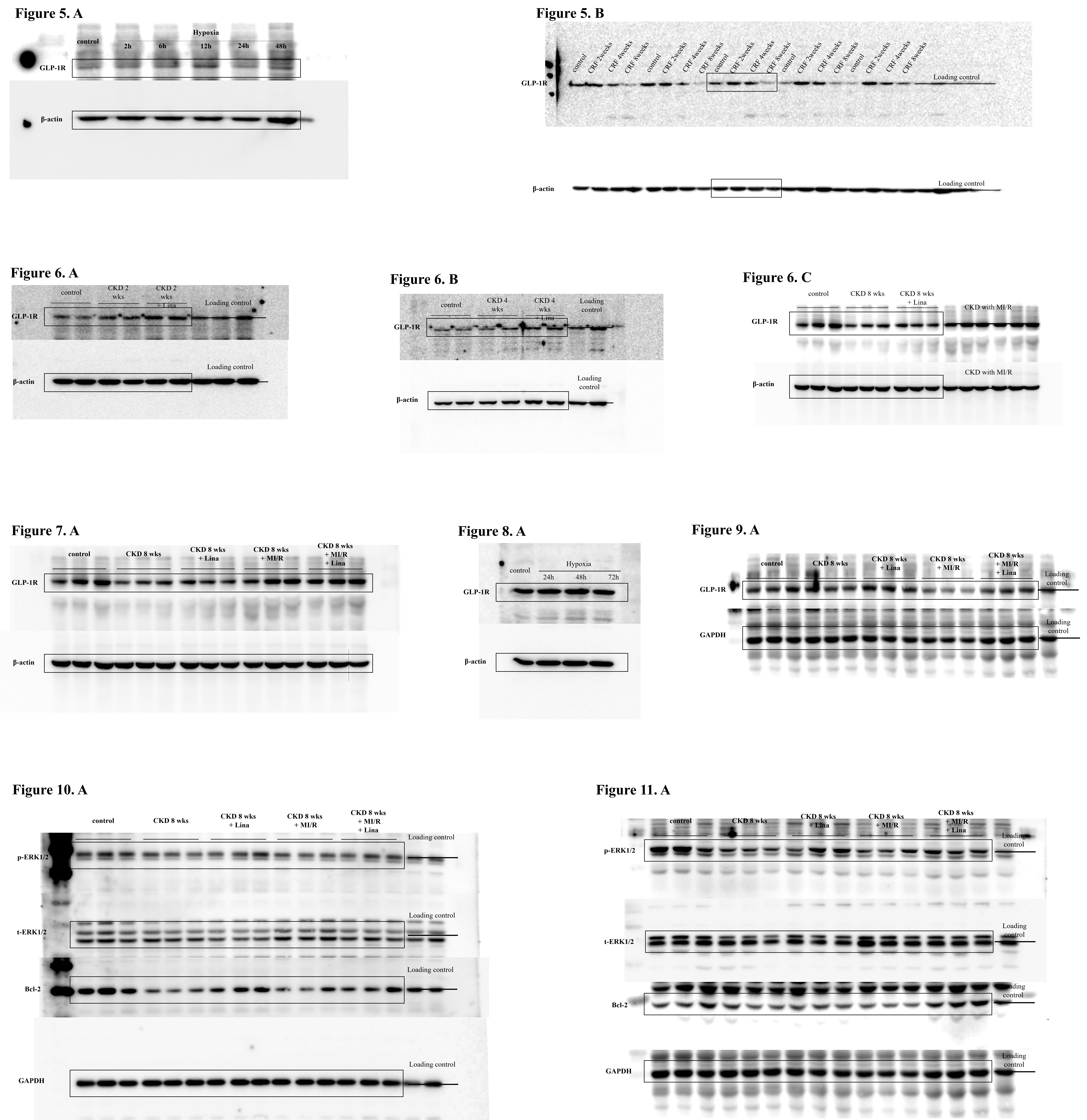

Supplement: Supplementary file 1 — Figure S1. Original blot of protein expression. Western blot bands including markers were imaged by a LAS-3000 imaging system (Fujifilm life science, Minato-ku, Tokyo). (TIF 2187 kb) [file 12882_2019_1243_MOESM1_ESM.tif]
